# Efficacy of cyclosporine combination therapy for new-onset minimal change nephrotic syndrome in adults

**DOI:** 10.1007/s10157-014-0975-0

**Published:** 2014-04-27

**Authors:** Akira Fujiwara, Nobuhito Hirawa, Yusuke Kobayashi, Keisuke Yatsu, Mari Katsumata, Yohsuke Ehara, Yuki Okuyama, Jun Yutoh, Tomoko Kaneda, Megumi Fujita, Yuichiro Yamamoto, Sanae Saka, Yoshiyuki Toya, Gen Yasuda, Satoshi Umemura

**Affiliations:** 1 Department of Medical Science and Cardiorenal Medicine, Yokohama City University School of Medicine, Yokohama, Japan; 2 Department of Nephrology and Hypertension, Yokohama City University Medical Center, 45-7 Urafune-cho, Minami-ku, Yokohama, 232-0024 Japan

**Keywords:** Cyclosporine, Minimal change nephrotic syndrome, Prednisolone, Clinical efficacy, Drug nephrotoxicity

## Abstract

**Background:**

Cyclosporine and prednisolone combination therapy has been used in the treatment of minimal change nephrotic syndrome (MCNS). However, few studies have evaluated the efficacy of cyclosporine combined with intravenous methylprednisolone pulse therapy (MPT) as a first-line treatment for new-onset MCNS. We conducted a retrospective clinical study to evaluate the efficacy and safety of cyclosporine combined with MPT and oral prednisolone for new-onset MCNS in adults.

**Methods:**

Forty-six adult patients with biopsy-proven MCNS were analyzed retrospectively. This study included three groups. Group 1 (*n* = 17) was treated with intravenous MPT (0.5 or 1.0 g/day for 3 days) followed by oral cyclosporine (2–3 mg/kg/day) and prednisolone (30 mg/day). Group 2 (*n* = 15) was treated with intravenous MPT followed by oral prednisolone (0.4–0.8 mg/kg/day). Group 3 (*n* = 14) was treated with oral prednisolone (0.6–1.0 mg/kg/day) alone.

**Results:**

The length of hospital stay was the shortest in Group 1 (*P* < 0.001). The mean duration to achieve <20 mg/day of prednisolone was also the shortest in Group 1 (*P* < 0.05). Complete remission rates were 100 % in Group 1, 85.7 % in Group 2, and 69.2 % in Group 3 during the 9-month follow-up (*P* = 0.073). The rate of adverse effects caused by prednisolone was less in Group 1 (*P* < 0.05). Multivariate analysis revealed that the independent determinants of durations of remission were the selectivity index (*P* = 0.004), eGFR (*P* = 0.001) and the use of cyclosporine (*P* = 0.045).

**Conclusions:**

Combination therapy with cyclosporine may be a beneficial treatment option for new-onset MCNS in adults because of its clinical efficacy and safety.

## Introduction

In the past several decades, prednisolone has been the most reliable treatment for minimal change nephrotic syndrome (MCNS). However, long-term steroid therapy readily induces adverse drug reactions such as diabetes mellitus, gastric complications, infections, osteoporosis, and psychiatric symptoms, which may compromise the quality of life (QOL) of patients. Furthermore, long periods of hospitalization for the treatment of nephrotic syndrome decrease the QOL of these patients. Thus, the length of hospital stay (LOS) should be shortened, and this is also desirable for the treatment of nephrotic syndrome from the viewpoint of medical economics. Intravenous methylprednisolone pulse therapy (MPT) followed by oral prednisolone has more recently become one of the treatments for intractable MCNS [1]. While this modality has been shown to improve remission rates, it still requires the long-term administration of a large amount of prednisolone.

Cyclosporine, an anti-T cell agent, has recently been considered as a more rational treatment than corticosteroids for MCNS, which is putatively associated with T cell abnormalities. Furthermore, cyclosporine acts not only as an anti-T cell agent, but also as a stabilizer for the actin cytoskeleton in kidney podocytes; therefore, it is beneficial for treating proteinuric kidney diseases [2]. Many studies have consequently focused on the efficacy of cyclosporine and prednisolone combination therapy in the treatment of intractable nephrotic syndromes. However, the most effective treatment option has yet to be elucidated. Therefore, we conducted a retrospective study to evaluate the effectiveness and safety of the major regimens used as first-line treatments for new-onset MCNS.

## Materials and methods

### Patient selection

Among patients with biopsy-proven new-onset MCNS admitted to our hospitals between 1996 and 2012, we selected patients who fulfilled the following criteria. First, they could be followed for at least 6 months after the initiation of treatment. Second, they had proteinuria in excess of 3.5 g/day and serum albumin concentrations of <3.0 g/dl at the start of treatment. Third, MCNS was diagnosed pathologically by light microscopic findings, and confirmed by negative immunofluorescence and typical ultrastructural morphology. Fourth, patients were not treated with corticosteroids or cytotoxic agents. This study was approved by the IRB/Ethics Committee of Yokohama City University Medical Center (D-1309006).

### Therapies and measurements

Three groups were included in the present study and were listed in Table 1. Pretreatment baseline parameters, including creatinine clearance, estimated glomerular filtration rate (eGFR), urinary protein excretion, serum total cholesterol concentration, serum albumin concentration, and serum hemoglobin concentration were measured. After discharge, blood pressure, urinary protein excretion, and serum creatinine levels were monitored on an outpatient basis every 2–4 weeks. The adverse effects of cyclosporine and prednisolone were monitored based on medical records. The selectivity index was calculated as the clearance of IgG divided by the clearance of transferrin. All patients were instructed to follow a low-sodium diet (5 g/day). Patients with marked edema were administered furosemide orally or intravenously, and few patients received intravenous albumin.

**Table 1:** Treatment groups

Group 1	Patients received cyclosporine (2–3 mg/kg/day) and intravenous methylprednisolone pulse therapy (0.5 or 1.0 g/day for 3 days), which were followed by the oral administration of prednisolone (initial doses 30 mg/day). The dose of cyclosporine was maintained at whole-blood trough levels between 50 and 150 ng/ml until the end of the first 6-month treatment period
Group 2	Patients received intravenous methylprednisolone pulse therapy (0.5 or 1.0 g/day for 3 days) followed by the oral administration of prednisolone (initial doses 0.4–0.8 mg/kg/day)
Group 3	Patients received oral prednisolone alone (initial doses 0.6–1.0 mg/kg/day)

### Definitions of remission

The response of treatment in nephrotic syndrome was categorized as complete remission, partial remission, or no response. Complete remission was defined as a reduction in proteinuria to below 300 mg/day for three consecutive days. Partial remission was defined as proteinuria of over 300 mg/day, but below 3.5 g/day. No response was defined as proteinuria of more than 3.5 g/day. The relapse of nephrotic syndrome was defined as proteinuria in excess of 1 g/day that lasted for more than three consecutive days during the follow-up.

### Assessment of clinical efficacy

The LOS after commencing the treatment to discharge, and to complete remission were evaluated as short-term effects. Long-term effects were assessed by the total amount of prednisolone, duration to achieve <20 mg/day of prednisolone, and duration of sustained remission (defined as no relapse). Major adverse effects caused by steroids, including diabetes mellitus, peptic ulcers, infections, bone fractures, and psychiatric symptoms were recorded. These adverse effects were defined by the following criteria: diabetes mellitus; use of anti-diabetic medication, peptic ulcer; based on positive endoscopic findings, infection; requiring medication, bone fracture; induced by steroids including vertebra fracture and femoral neck fracture, psychiatric symptoms; requiring medication, and hypertension; systolic blood pressure >140 mmHg, diastolic blood pressure >90 mmHg or the initiation of antihypertensive medication.

### Statistical analysis

Data are expressed as the mean ± standard deviation. Statistical analyses were performed using a one-way analysis of variance (ANOVA) followed by Tukey’s post hoc test. Chi-squared tests were used for comparisons between categorical variables. Remission curves were evaluated by Kaplan–Meier method. A possible predictor of the LOS after the treatment, durations of remission, and major adverse effects were tested by multivariate analysis. Statistical analyses were performed using SPSS statistics 19 (IBM) or Stat-View J version 5.0 (SAS institute Inc). Values of *P* < 0.05 were considered significant.

## Results

### Patient characteristics

From 53 patients with MCNS identified in the initial screening, we selected 46 patients who fulfilled the inclusion criteria of this study and divided them into three groups according to the treatment regimen. The clinical characteristics of patients in the three groups are shown in Table 2. No significant differences were observed in any of the parameters examined. The mean dose of cyclosporine required to maintain the whole-blood trough level between 50 and 150 ng/ml was 118 ± 30 mg/day (range 50 and 200 mg/day) during the first 6 months of treatment. The average doses of prednisolone initiated immediately after MPT were 30.0 ± 0.0 and 39.0 ± 6.3 mg/day in Groups 1 and 2, respectively. The initial dose of prednisolone in Group 3 was 47.9 ± 7.0 mg/day. The dose of prednisolone was tapered by 5–10 mg every 4–8 weeks. No significant differences were observed in the average doses of prednisolone at discharge among three groups (27.9 ± 3.6 mg/day in Group 1; 30.7 ± 4.6 mg/day in Group 2; 30.4 ± 1.3 mg/day in Group 3; *P* = 0.062).

**Table 2:** Patients characteristics

Characteristic	Group 1 (*n* = 17)	Group 2 (*n* = 15)	Group 3 (*n* = 14)	*P* value
Age at diagnosis (years)	37 ± 18	37 ± 16	39 ± 19	0.949
Sex (male:female)	8:9	9:6	9:5	0.596
Body mass index	25.2 ± 5.1	23.7 ± 3.2	22.7 ± 3.4	0.247
Selectivity index	0.12 ± 0.05	0.13 ± 0.10	0.13 ± 0.05	0.890
Systolic blood pressure (mmHg)	119 ± 17	120 ± 17	122 ± 13	0.866
Diastolic blood pressure (mmHg)	73 ± 11	78 ± 11	74 ± 11	0.419
Body weight (kg)	67 ± 17	65 ± 13	63 ± 13	0.722
Creatinine clearance (ml/min)	88 ± 42	88 ± 34	91 ± 42	0.966
eGFR (ml/min/1.73 m^2^)	67 ± 22	73 ± 26	74 ± 25	0.899
Urinary protein excretion (g/day)	7.8 ± 3.9	11.3 ± 6.1	7.9 ± 4.5	0.095
Total cholesterol (mg/dl)	488 ± 194	581 ± 284	492 ± 109	0.392
Albumin (g/dl)	1.6 ± 0.5	1.6 ± 0.6	2.0 ± 0.6	0.059
Hemoglobin (g/dl)	14.9 ± 1.7	15.2 ± 1.7	15.1 ± 2.5	0.933

*eGFR* estimated glomerular filtration rate

### Days of hospitalization

**Fig. 1:**
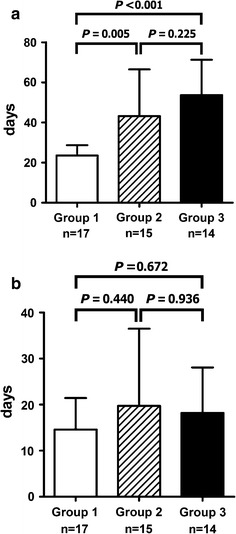
Length of hospital stay (**a**) and days required to attain complete remission (**b**) after the start of therapy in the three groups

The LOS after the start of therapy was the shortest in Group 1 and the longest in Group 3 (23.6 ± 5.1 days in Group 1; 43.2 ± 23.3 days in Group 2; 53.6 ± 17.6 days in Group 3, *P* < 0.001 by ANOVA, Fig. 1a).

### Durations of remission

All patients achieved complete remission at 10 weeks. No significant differences were observed in the mean durations to enter complete remission after the start of therapy among the three groups (14.6 ± 6.9 days in Group 1; 19.7 ± 16.8 days in Group 2; 18.2 ± 9.9 days in Group 3; *P* = 0.450 by ANOVA, Fig. 1b).

### Total amount of prednisolone used

**Fig. 2:**
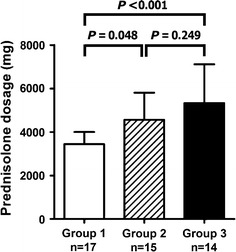
Total amount of prednisolone administered during therapy for 6 months in the three groups

The total amount of prednisolone used after the start of therapy to 6 months was the smallest in Group 1 and highest in Group 3 (3,444 ± 559 mg in Group 1; 4,558 ± 1,251 mg in Group 2; 5,330 ± 1,333 mg in Group 3; *P* < 0.001 by ANOVA, Fig. 2). The total amounts of oral prednisolone and methylprednisolone were similar in Groups 1 and 3 at 6 months.

### Duration to achieve less than 20 mg/day of prednisolone

**Fig. 3:**
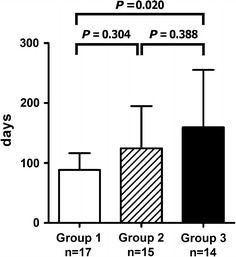
Days required to achieve <20 mg/day of prednisolone after the start of therapy in the three groups

The mean duration to achieve <20 mg/day of prednisolone after the start of therapy was the shortest in Group 1 and the longest in Group 3 (88.5 ± 28.0 days in Group 1; 124.5 ± 70.4 days in Group 2; 159.4 ± 96.0 days in Group 3, *P* = 0.026 by ANOVA, Fig. 3).

### Relapse rate

**Fig. 4:**
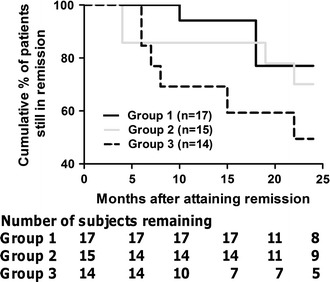
Duration of sustained remission in the three groups. The proportion of patients who remained in remission during the subsequent 24 months was calculated by the life-table method

Figure 4 shows the duration of sustained remission analyzed by the life-table method. During a follow-up period of 9 months, Group 1 showed no relapse and maintained a remission rate of 100 %, whereas Groups 2 and 3 had remission rates of 85.7 and 69.2 %, respectively (*P* = 0.073). The estimated sustained remission rate at 24 months was 77 % in Group 1, 70 % in Group 2, and 49 % in Group 3 (*P* = 0.226).

### Renal function

No significant differences were observed in average serum creatinine levels between 6 months after the start of therapy and prior to the treatment in all groups (Group 1: 1.02 ± 0.48–0.83 ± 0.14 mg/dl, *P* = 0.135; Group 2: 0.97 ± 0.41–0.81 ± 0.23 mg/dl, *P* = 0.064; Group 3: 0.95 ± 0.31–0.82 ± 0.18 mg/dl, *P* = 0.120). Similarly, the average eGFR 6 months after the start of treatment was not significantly different from the pretreatment baseline level in all groups (Group 1: 70.0 ± 22.4–78.1 ± 17.1 ml/min/1.73 m^2^, *P* = 0.210; Group 2: 72.6 ± 26.2–79.3 ± 22.0 ml/min/1.73 m^2^, *P* = 0.083; Group 3: 73.9 ± 24.7–81.2 ± 31.3 ml/min/1.73 m^2^, *P* = 0.245). No patient in any group developed renal dysfunction.

### Adverse effects

The adverse effects observed during the 6 months following the start of therapy are summarized in Table 3. The rates of steroid-induced major adverse effects were significantly lower (*P* = 0.042) in Group 1. The incidence of new-onset hypertension was 12.5 % (2/16) in Group 1, 7.7 % (1/13) in Group 2, and 8.3 % (1/12) in Group 3 6 months after the start of therapy with no significant difference (*P* = 0.851).

**Table 3:** Major adverse effects caused by prednisolone during the 6 months following the start of therapy

Adverse effects	Group 1 (*n* = 17)	Group 2 (*n* = 15)	Group 3 (*n* = 14)
Diabetes mellitus	0	3	3
Peptic ulcer	0	0	2
Infection	0	3	1
Bone fracture	0	0	1
Psychiatric symptoms	2	2	0

### Medical costs

Because the LOS was shortened, the total medical cost in Group 1 was significantly lower than that in Group 3 after the start of therapy to discharge (*P* < 0.001).

### Multivariate analysis

We assessed correlations using multivariate analysis. The independent determinants of the LOS after treatments were the selectivity index and the use of cyclosporine; and the independent determinants of the durations of remission were the selectivity index, eGFR, and the use of cyclosporine, as shown in Table 4. The adverse effects were negatively associated with the use of cyclosporine (*P* = 0.001).

**Table 4:** Multivariate analysis to assess correlations with other variables in all subjects

Variable	LOS after the treatment			Durations of remission		
	Regression coefficient	*T* value	*P* value	Regression coefficient	*T* value	*P* value
Age	−0.069	−0.579	0.566	−0.217	−1.683	0.101
eGFR	−0.249	−1.937	0.060	−**0.483**	−**3.466**	**0.001**
Urinary protein excretion	−0.138	−1.144	0.260	−0.115	0.878	0.386
Serum albumin	0.049	0.392	0.698	−0.047	−0.345	0.732
Selectivity index	**0.384**	**3.374**	**0.002**	**0.377**	**3.051**	**0.004**
Use of cyclosporine	−**0.607**	−**5.803**	**<0.001**	−**0.235**	−**2.069**	**0.045**

Bold values are statistically significant

*LOS* length of hospital stay, *eGFR* estimated glomerular filtration rate

## Discussion

Although steroid therapy has been the standard treatment for MCNS, 30–70 % of patients with adult-onset MCNS treated with prednisolone monotherapy have frequent relapses and develop steroid dependence or resistance [3, 4]. MPT was subsequently established and shown to rapidly induce remission even in idiopathic steroid-resistant nephrotic syndrome (SRNS) [5]. However, whether MPT followed by low-dose prednisolone therapy (0.5 mg/kg/day) is superior to high-dose prednisolone monotherapy (1 mg/kg/day) remains unclear [1, 6]. Another therapeutic regimen combining prednisolone with cyclosporine has more recently been examined in MCNS patients. Eguchi et al. [7] showed that the co-administration of cyclosporine and prednisolone (0.8 mg/kg/day) to adult patients with the first relapse of MCNS significantly reduced the time to remission and allowed the prednisolone dose to be reduced more than that with prednisolone monotherapy (1.0 mg/kg/day). Matsumoto et al. [8] demonstrated that cyclosporine (2–3 mg/kg/day) after MPT was not only advantageous for the rapid induction of complete remission, but was efficient for maintaining remission with little evidence of cyclosporine toxicity in adult patients with the relapse or the first episode of MCNS. Hamasaki et al. [9] showed that cyclosporine in combination with prednisolone induced higher complete remission rates than prednisolone monotherapy in children with steroid-resistant MCNS or other types of nephrotic syndrome. Thus, cyclosporine combined with MPT may further improve clinical efficacy and safety.

According to the guidelines of KDIGO for glomerulonephritis, corticosteroids are recommended as an initial treatment of MCNS in adults with evidence level 1C [10]. However, these treatments require long periods of hospitalization. As shown in our study, the mean LOS in Group 3 was 53.6 days. The long period of hospitalization has been shown to markedly reduce the QOL of the adult patients [11]. On the other hand, the guidelines of KDIGO for glomerulonephritis and workshop recommendations for cyclosporine described the usefulness of cyclosporine in steroid-resistant MCNS [10, 12]. Cyclosporine was additionally used for the treatment of MCNS in order to induce sustained remission in some cases. Several other studies have suggested that the long-term maintenance treatment of MCNS with cyclosporine may be efficient and safe at least for a period of up to a few years [13]. In the present study, we attempted to clarify whether cyclosporine combination therapy could lead to the rapid induction of remission and/or shorten hospitalization without severe adverse effects in MCNS adult patients. The administration of cyclosporine to children for the initial treatment of MCNS has been reported previously [14]. However, few studies have been conducted on adults. Our results clearly showed the benefits of cyclosporine with prednisolone in shortening the LOS without increasing the rate of adverse effects. Furthermore, this treatment protocol decreased the amount of prednisolone used and medical costs. Multivariate analysis revealed that the durations of remission correlated with cyclosporine treatment, which indicated that the cyclosporine treatment has benefits in reducing the LOS and also partly shortening the periods to complete remission.

The incidence of refractory nephrotic syndrome is higher in the elderly, and MCNS accounts for ~10 % of all cases of nephrotic syndrome in this population. However, the characteristics of MCNS in the elderly have not yet been established [15]. Older adult patients (>50 years) and younger patients (18–50 years) with MCNS administered oral prednisolone (0.8 mg/kg/day) were shown to have similar steroid responsiveness, whereas older patients were at higher risk of acute kidney injury [16, 17]. Each of the three treatment groups in our study had 4 older patients (mean age; 64 vs. 60 vs. 65 years old in Groups 1, 2, and 3, respectively). The periods from the start of the therapy to complete remission were shorter due to the cyclosporine treatment (14.5 vs. 19.5 vs. 22.0 days). Adverse effects were observed in 25 % of Group 1, 75 % of Group 2, and 75 % of Group 3. Furthermore, no relapse was reported within 12 months in Group 1 only. Thus, the combination of cyclosporine and prednisolone with intravenous MPT was also effective and safe in older patients.

Serious adverse effects caused by long-term steroid therapy are unavoidable in the treatment of MCNS adult patients. In the present study, more oral prednisolone was administered to Groups 2 and 3 than to Group 1. The rate of adverse effects caused by corticosteroids was also higher in these two groups than in Group 1. Thus, the additional administration of cyclosporine should have steroid-sparing effects to minimize the adverse effects caused by steroids. Cyclosporine causes its own specific adverse effects, including nephrotoxicity, hypertension, hepatotoxicity, and encephalopathy. Cyclosporine nephrotoxicity has been shown to correlate with the duration of heavy proteinuria and cyclosporine doses [18, 19]. No significant differences were observed in the development of hypertension or changes in eGFR and serum creatinine levels among the three groups. The dose of cyclosporine in Group 1 that showed trough levels between 50 and 150 ng/ml was almost half of that recommended in renal transplantation [20]. Thus, the lower doses of cyclosporine administered in this study may explain why cyclosporine caused minimum adverse effects and mild reductions in prednisolone doses.

MPT was used to improve the efficacy of the prednisolone treatment and decrease the adverse effects of prednisolone due to the lower doses administered as a maintenance therapy. The total amounts of oral prednisolone and methylprednisolone were similar in Groups 1 and 3 at 6 months. However, the rate of adverse effects in Group 1 was lower than that in Group 3 in the present study. The adverse effects of prednisolone have been associated with the oral dose and administration period of high doses of prednisolone. An equal or more than 20 mg oral dose of prednisolone has been identified as a risk factor for fractures, infections, and gastric ulcers [21, 22]. Thus, we further calculated and compared the administration periods of orally administered prednisolone of 20 mg and more in our study. The administration period of 20 mg/day or more of prednisolone was the shortest in Group 1. Under these conditions, we further analyzed relationships between adverse effects and various factors, including the use of cyclosporine. Multivariate analysis revealed that the independent determinants of adverse effects were negatively correlated with the usage of cyclosporine, only. Thus, cyclosporine treatments correlated with decreases in the rates of adverse effects.

Patients with MCNS typically stay for months in hospitals for their treatment. Medical expenses have always been a major issue for long-term hospitalization. There is very limited literature on the costs associated with SRNS in children and MCNS in adults. Colquitt et al. [23] showed the cost-effectiveness of treatments for children with idiopathic SRNS. The results of the present study suggest that combination therapy with cyclosporine has the advantages of shortening hospitalization and reducing adverse effects. These benefits may contribute to reductions in medical expenses.

Our study has some limitations. First, the total number of patients was small in the retrospective study, which could be a source of selection bias. The treatment protocol for Group 1 is the latest treatment option, and we asked this treatment for all patients who met the study criteria. The treatment protocol for Group 2 and Group 3 were freely chosen by the doctor in charge. However, no significant differences were observed in baseline parameters among the three groups. Thus, selection bias may be minimal. Second, repeated kidney biopsies are required to evaluate renal function and adverse effects during long-term treatment. Third, edema in the intestine has been reported in patients with severe nephrotic syndrome, and this may decrease the absorption of drugs, including prednisolone [24]. Thus, intravenous MPT was adopted as the treatment of choice. As the treatment benefits were limited in the intravenous MPT (Group 2) compared to the prednisolone monotherapy (Group 3) in the present study, we consider combined cyclosporine and oral prednisolone therapy without MPT might be a potential treatment for new-onset MCNS in adults.

In conclusion, cyclosporine combined with MPT and oral prednisolone shortened the LOS and decreased the total amount of prednisolone without severe adverse effects when used in patients with the first attack of adult-onset MCNS. Although no significant differences were observed in the days required for complete remission among the three groups, cyclosporine use was associated with the period to complete response in multivariate analysis, and relapse rates were slightly lower in Group 1 than in Group 3. Combination therapy with cyclosporine may be a useful treatment option currently available for new-onset MCNS in adults.

## References

[1] Imbasciati E, Gusmano R, Edefonti A, Zucchelli P, Pozzi C, Grassi C (1985). Controlled trial of methylprednisolone pulses and low dose oral prednisone for the minimal change nephrotic syndrome. Br Med J (Clin Res Ed)..

[2] Faul C, Donnelly M, Merscher-Gomez S, Chang YH, Franz S, Delfgaauw J (2008). The actin cytoskeleton of kidney podocytes is a direct target of the antiproteinuric effect of cyclosporine A. Nat Med..

[3] Takei T, Koike M, Suzuki K, Shirota S, Itabashi M, Ohtsubo S (2007). The characteristics of relapse in adult-onset minimal-change nephrotic syndrome. Clin Exp Nephrol..

[4] Nakayama M, Katafuchi R, Yanase T, Ikeda K, Tanaka H, Fujimi S (2002). Steroid responsiveness and frequency of relapse in adult-onset minimal change nephrotic syndrome. Am J Kidney Dis..

[5] Yorgin PD, Krasher J, Al-Uzri AY (2001). Pulse methylprednisolone treatment of idiopathic steroid-resistant nephrotic syndrome. Pediatr Nephrol..

[6] Fukudome K, Fujimoto S, Sato Y, Kitamura K (2012). Comparison of the effects of intravenous methylprednisolone pulse versus oral prednisolone therapies on the first attack of minimal-change nephrotic syndrome in adults. Nephrology..

[7] Eguchi A, Takei T, Yoshida T, Tsuchiya K, Nitta K (2010). Combined cyclosporine and prednisolone therapy in adult patients with the first relapse of minimal-change nephrotic syndrome. Nephrol Dial Transplant..

[8] Matsumoto H, Nakao T, Okada T, Nagaoka Y, Takeguchi F, Tomaru R (2004). Favorable outcome of low-dose cyclosporine after pulse methylprednisolone in Japanese adult minimal-change nephrotic syndrome. Intern Med..

[9] Hamasaki Y, Yoshikawa N, Hattori S, Sasaki S, Iijima K, Nakanishi K (2009). Cyclosporine and steroid therapy in children with steroid-resistant nephrotic syndrome. Pediatr Nephrol..

[10] Radhakrishnan J, Cattran DC (2012). The KDIGO practice guideline on glomerulonephritis: reading between the (guide)lines–application to the individual patient. Kidney Int..

[11] DeOreo PB (1997). Hemodialysis patient-assessed functional health status predicts continued survival, hospitalization, and dialysis-attendance compliance. Am J Kidney Dis..

[12] Cattran DC, Alexopoulos E, Heering P, Hoyer PF, Johnston A, Meyrier A (2007). Cyclosporin in idiopathic glomerular disease associated with the nephrotic syndrome: workshop recommendations. Kidney Int..

[13] Meyrier A, Noel LH, Auriche P, Callard P (1994). Long-term renal tolerance of cyclosporin A treatment in adult idiopathic nephrotic syndrome. Collaborative Group of the Societe de Nephrologie. Kidney Int..

[14] Tejani A, Suthanthiran M, Pomrantz A (1991). A randomized controlled trial of low-dose prednisone and ciclosporin versus high-dose prednisone in nephrotic syndrome of children. Nephron..

[15] Mori D, Shinzawa M, Namba T, Yamaguchi Y, Itano S, Imakita N (2012). Clinical characteristics of adult-onset minimal change nephrotic syndrome in our hospital. Jpn J Nephrol..

[16] Tse KC, Lam MF, Yip PS, Li FK, Choy BY, Lai KN (2003). Idiopathic minimal change nephrotic syndrome in older adults: steroid responsiveness and pattern of relapses. Nephrol Dial Transplant..

[17] Waldman M, Crew RJ, Valeri A, Busch J, Stokes B, Markowitz G (2007). Adult minimal-change disease: clinical characteristics, treatment, and outcomes. Clin J Am Soc Nephrol..

[18] Iijima K, Hamahira K, Tanaka R, Kobayashi A, Nozu K, Nakamura H (2002). Risk factors for cyclosporine-induced tubulointerstitial lesions in children with minimal change nephrotic syndrome. Kidney Int..

[19] Kengne-Wafo S, Massella L, Diomedi-Camassei F, Gianviti A, Vivarelli M, Greco M (2009). Risk factors for cyclosporin A nephrotoxicity in children with steroid-dependant nephrotic syndrome. Clin J Am Soc Nephrol..

[20] Kidney Disease: Improving Global Outcomes Transplant Work G (2009). KDIGO clinical practice guideline for the care of kidney transplant recipients. Am J Transplant..

[21] Summey BT, Yosipovitch G (2006). Glucocorticoid-induced bone loss in dermatologic patients: an update. Arch Dermatol..

[22] Ferrante M, D’Hoore A, Vermeire S, Declerck S, Noman M, Van Assche G (2009). Corticosteroids but not infliximab increase short-term postoperative infectious complications in patients with ulcerative colitis. Inflamm Bowel Dis..

[23] Colquitt JL, Kirby J, Green C, Cooper K, Trompeter RS (2007). The clinical effectiveness and cost-effectiveness of treatments for children with idiopathic steroid-resistant nephrotic syndrome: a systematic review. Health Technol Assess..

[24] Matsuo S, Imai E, Saito T, Taguchi T, Yokoyama H, Narita I (2011). Guidelines for the treatment of nephrotic syndrome. Jpn J Nephrol..

